# Clinicopathological Characteristics and Prognosis of Upper Gastric Cancer Patients in China: A 32-Year Single-Center Retrospective Clinical Study

**DOI:** 10.1155/2019/9248394

**Published:** 2019-12-01

**Authors:** Yingbo Gong, Pengliang Wang, Zhi Zhu, Junyan Zhang, Jinyu Huang, Huimian Xu

**Affiliations:** ^1^Department of Surgical Oncology, The First Hospital of China Medical University, 155 Nanjing North Street, Heping District, Shenyang 110001, China; ^2^Key Laboratory of Gastric Cancer Molecular Pathology of Liaoning Province, 155 Nanjing North Street, Heping District, Shenyang 110001, China

## Abstract

**Purpose:**

Upper or proximal gastric cancer occurs in the upper third of the stomach between the cardia and a line connecting the greater and lesser curvatures. As it differs from other gastric cancers in pathology and prognosis, we evaluated patient and disease characteristics that might guide improved treatment and survival of upper gastric cancer.

**Methods:**

We conducted a retrospective analysis of 649 patients with upper gastric cancer and 1551 patients with lower gastric cancer and R0 radical surgery at our institution between January 1980 and December 2012.

**Results:**

Survival after radical surgery for upper gastric cancer was 77.8% at 1 year, 49.6% at 3 years, and 41.1% at 5 years. The corresponding rates for lower gastric cancer were 85.9%, 60.0%, and 57.2% (*p* < 0.001). Upper gastric cancer had a poor prognosis. Sex (*p* = 0.036), tumor diameter (*p* = 0.001), macroscopic type (*p* < 0.001), pTM stage (*p* < 0.001), tissue differentiation type (*p* = 0.003), and serosal invasion (*p* = 0.034) were independently associated with lymph node metastasis. The macroscopic type (*p* = 0.045), lymphovascular tumor emboli (*p* = 0.021), and pTNM stage were independently associated with recurrence and metastasis. Survival of 333 patients with D2 total gastrectomy was 81.3% at 1 year, 54.4% at 3 years, and 45.2% at 5 years. The corresponding rates for 316 proximal gastrectomy patients were 75.4%, 44.9%, and 36.7%. Radical total gastrectomy had better survival than radical proximal resection.

**Conclusions:**

Upper gastric cancers were more aggressive, had a worse prognosis, and were more prone to recurrence and metastasis compared with lower gastric cancers. Survival was better after total gastrectomy than after proximal resection.

## 1. Background

Gastric cancer is the sixth most prevalent malignant tumor worldwide and the third leading cause of cancer-related deaths. The International Agency for Research on Cancer estimated that there were about one million new cases of gastric cancer and 782685 deaths from gastric cancer in 2018 [1]. Upper gastric cancer, also known as proximal gastric cancer, occurs below the cardia and above a line connecting the greater and lesser curvatures, and its incidence is increasing [2–5]. The epidemiology, pathology, surgical treatment, and prognosis of upper gastric cancer differ from those of lower stomach cancer. Its anatomical location makes upper stomach cancer relatively difficult to find, and it lacks specific symptoms at early stages of disease. Diagnosis at a mid or late stage increases the difficulty of treatment and leads to a poor prognosis [4, 6, 7]. An evaluation of dysphagia may lead to an early diagnosis [8, 9]. At present, the choice of surgical treatment has changed from simple anatomy to oncology biology, immunology, and anatomy. The clinical and pathological characteristics of individual upper gastric cancer patients may be of value in choosing surgical options that will improve survival. Radical surgery is currently the most effective treatment, but the extent of lymph node dissection and the optimal surgical procedure may not be easy to determine [10–12]. The objective of this retrospective analysis of patient characteristics, surgical treatment, and cancer prognosis was to identify clinical, pathological, and surgical variables that were associated with improved outcomes, prognosis, and survival of those with upper gastric cancer.

## 2. Patients and Methods

### 2.1. Patient Selection and Study Design

A group of 2200 patients with radical surgery for gastric cancer at The First Hospital of China Medical University, Shenyang, Liaoning, China, between January 1980 and December 2012 were retrospectively evaluated. Patients with a postoperative pathological diagnosis of primary gastric adenocarcinoma, no other history of gastric surgery, no neoadjuvant therapy before surgery, and no preoperative imaging evidence of distant metastasis were eligible. All surgeries were R0 resections; postoperative pathology was evaluated by two experienced pathologists. The lymph node grouping, transfer site, and numbers were included in the pathology report. All included patients had complete follow-up data, and there was no loss of follow-up. Of the 2200 included patients, 649 had upper gastric cancer and 1551 had lower gastric cancer.

### 2.2. Pathological Criteria

The macroscopic pathology included Borrmann types 1 and 2 for local and Borrmann types 3 and 4 for infiltrative disease. Tissue growth was classified as agglomerate, nest, or diffuse. Tissue typing included highly and moderately differentiated papillary adenocarcinoma and well- and poorly differentiated tubular adenocarcinoma. Signet ring cell carcinoma, mucinous adenocarcinoma, and undifferentiated carcinoma were classified as poorly differentiated. The depth of tumor invasion involving the mucosa, submucosa, muscularis propria, subserosa, and the serosal surface and lymph node metastasis were classified following the 14^th^ edition of the Gastric Cancer Treatment Protocol of the Japanese Gastric Cancer Association. Pathological staging followed the eighth edition of the American Joint Committee on Cancer tumor, node, and metastasis (TNM) staging system.

### 2.3. Statistical Analysis

Count data were analyzed by chi-squared tests and logistic regression. Cumulative survival was analyzed and plotted by the Kaplan-Meier method; differences were evaluated with log-rank tests. Cox proportional hazard regression was used for multifactor analysis. SPSS 23.0 (IBM Corp., Armonk, NY, USA) was used for the statistical analysis, and Microsoft Excel was used for data collation. *p* < 0.05 was considered statistically significant.

## 3. Results

### 3.1. Clinicopathological Characteristics of Upper and Lower Gastric Cancers

Patient characteristics are shown in Table 1. The ratio of male to female patients was significantly higher in upper than in lower gastric cancer patients (4.5 : 1 versus 2.4 : 1, *p* < 0.001). The average age of upper gastric cancer patients was 61.5 years compared with 57.5 years in lower gastric cancer patients (*p* = 0.003). Three hundred and eighty-two (58.9%) of upper gastric cancers have ≥5 cm diameter compared with 605 (39%) of the lower gastric cancers (*p* < 0.001). Diagnosis was at an early stage in 47 (7.2%) of upper gastric cancer patients and 332 (21.4%, *p* < 0.001) of lower gastric cancer patients. The pT stages and numbers of positive lymph nodes are shown in Table 1. Fewer patients with upper (35.7%) than lower (41.2%) gastric cancer were lymph node negative (*p* = 0.046). The degree of differentiation of upper cancer tissues was significantly less than that of lower gastric cancers (*p* = 0.046). The growth patterns of upper and lower gastric cancers were significantly different (*p* < 0.001). Lymphovascular tumor emboli occurred in 172 of upper (26.5%) and 346 of lower (22.3%) gastric cancer tumors. Serosal invasion occurred in 264 of upper (40.7%) and 375 of lower (24.2%) gastric cancers (*p* < 0.001). Recurrence and metastasis occurred in 277 cases of upper (42.7%) and 526 cases of lower (33.9%) gastric cancer (*p* < 0.001).

### 3.2. Prognosis of Upper Gastric Cancer

**Figure 1:**
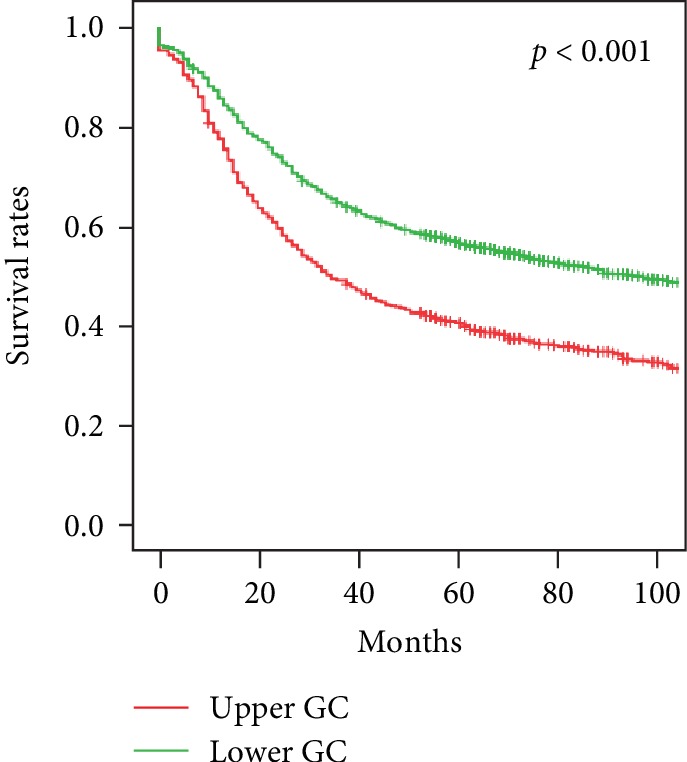
Survival of patients with upper and lower gastric cancers, *p* < 0.001. Abbreviations: GC: gastric cancer.

**Figure 2:**
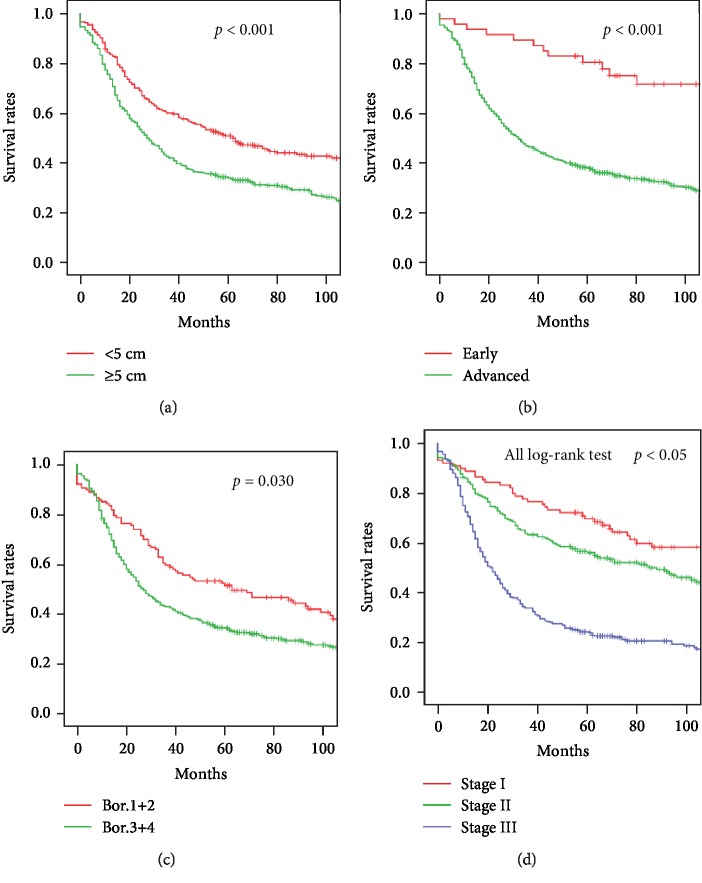
Survival of patients with upper gastric cancer: (a) tumor diameter, *p* < 0.001; (b) macroscopic type, *p* < 0.001; (c) Borrmann type, *p* = 0.030; (d) TNM stage, all log-rank test *p* < 0.05.

**Figure 3:**
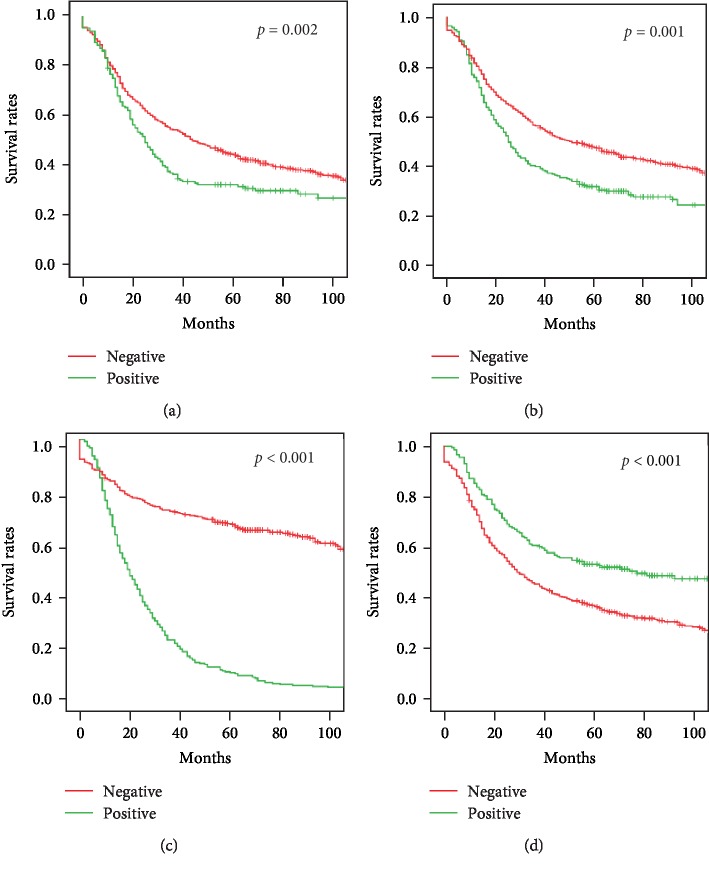
Survival of patients with upper gastric cancer: (a) lymphovascular tumor emboli, *p* = 0.002; (b) serosal invasion, *p* = 0.001; (c) recurrence and metastasis, *p* < 0.001; (d) adjuvant chemotherapy, *p* < 0.001.

The 1-year survival after radical surgery for upper gastric cancer was 77.8%, 3-year survival was 49.6%, and 5-year survival was 41.1%. The 1-year survival after surgery for lower gastric cancer was 85.9%, the 3-year survival was 60.0%, and the 5-year survival was 57.2% (*p* < 0.001, Figure 1). Univariate analysis showed that the tumor diameter, macroscopic type, pTNM stage, lymphovascular tumor embolus, serosal invasion, recurrence and metastasis, and postoperative adjuvant chemotherapy were associated with upper gastric cancer prognosis. Survival decreased with pTNM stage local Borrmann type 1 and 2 tumors, smaller tumor diameter, no lymphovascular tumor emboli, no serosal invasion, and no recurrence and/or metastasis. Survival was longer in patients with adjuvant chemotherapy after surgery (Figures 2 and 3). After multivariate analysis, the macroscopic tumor type, recurrence and metastasis, postoperative adjuvant chemotherapy, and pTNM staging were independently associated with upper gastric cancer prognosis (Table 2).

The characteristics of patients with and without lymph node metastasis are shown in Table 3. Metastatic lymph nodes were not found in 231 of the 649 patients with upper gastric cancer (35.6%); 418 (64.4%) had lymph node metastasis. The male : female ratio was 6.7 : 1 in the group without and 3.75 : 1 in the group with lymph node metastasis (*p* = 0.011). Lymph node metastasis occurred in 72.5% of patients with tumors ≥ 5 cm in diameter and in 52.8% of those with tumors < 5 cm in diameter (*p* < 0.001); in 68.9% of patients with poorly differentiated tumors and in 58.1% of patients with well-differentiated tumors (*p* = 0.005); in 17.0% of early cancers and in 68.1% of advanced cancers (*p* < 0.001); and in 48.4% of patients with macroscopic local tumors and in 73.3% of infiltrative tumors (*p* < 0.001). Lymph node metastasis increased significantly with pTNM staging (*p* < 0.001). The pN rates are shown in Table 3 and ranged from 17.0% for pT1 to 79.5% for pT4b tumors. Lymph node metastasis occurred in 75.8% of patients with serosal invasion and in 56.6% of those with no serosal invasion. Patient sex, age, tissue growth mode, and lymphovascular cancer embolus were not significantly different in patients with or without lymph node metastasis. Multivariate analysis and logistic regression (Table 4) revealed that sex (*p* = 0.036), tumor diameter (*p* = 0.001), macroscopic type (*p* < 0.001), pT stage (*p* < 0.001), tissue differentiation type (*p* = 0.003), and serosal invasion (*p* = 0.034) were independently associated with lymph node metastasis of upper gastric cancer.

**Figure 4:**
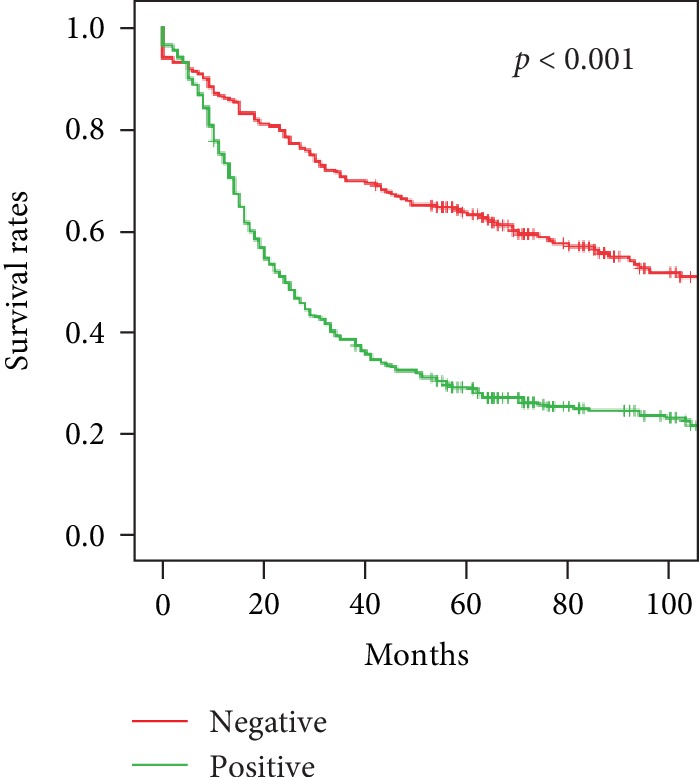
Survival of upper gastric cancer patients with and without lymph node metastasis, *p* < 0.001.

The survival analysis of patients with and without lymph node metastasis is shown in Figure 4. The 1-year survival of the 231 patients with no lymph node metastasis was 86.1%, 3-year survival was 69.7%, and 5-year survival was 63%. The corresponding survival rates for the 418 patients with lymph node metastasis were 73.2%, 38.4%, and 28.7% (*p* = 0.001). When pN stage was included in the analysis, 5-year survival ranged from 63.0% for pN0 to 11.3% for pN3b tumors (*p* < 0.001) showing that survival decreased with increased pN stage. Five-year survival was ranged from 79% for pT1 to 22.2% for pT4b stage patients without metastatic lymph nodes. Five-year survival of patients with corresponding pT stages and metastatic lymph nodes ranged from 65.6% to 8.6%.

### 3.3. Postoperative Recurrence and Metastasis of Upper Gastric Cancer

Tumor recurrence and metastasis occurred in 277 of the 649 patients with upper gastric cancer (42.7%). Recurrence occurred in 47.4% of patients with tumors ≥ 5 cm in diameter and in 36.0% of those with tumors < 5 cm (*p* = 0.004). Recurrence and metastasis occurred in 12.8% of early upper gastric cancers and 45.0% of advanced stage cancers (*p* < 0.001); 28.6% of localized tumors and 49.4% of infiltrating tumors (*p* < 0.001); 52.3% of patients with lymphovascular cancer emboli and 39.2% who were thrombus-negative (39.2%, *p* = 0.003); and 50% of patients with serosal invasion compared with 37.7% of those with noninvasive tumors (*p* = 0.002). As shown in Table 5, recurrence and metastasis increased significantly with pTNM stage, 17.8% at stage I, 27.1% at stage II, and 58.8% at stage III (*p* < 0.001). Multivariate analysis (Table 6) found that the macroscopic type (*p* = 0.045), lymphovascular tumor emboli (*p* = 0.021), and pTNM stage were independently associated with recurrence and metastasis after tumor resection.

### 3.4. Surgical Methods and Prognosis of Upper Gastric Cancer

**Figure 5:**
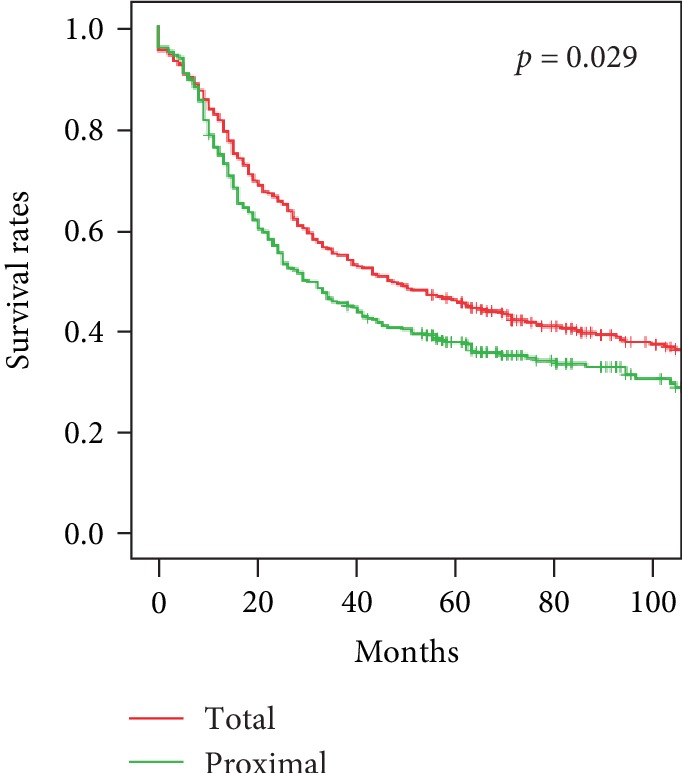
Survival of upper gastric cancer patients treated by total or proximal gastrectomy, *p* = 0.029.

**Figure 6:**
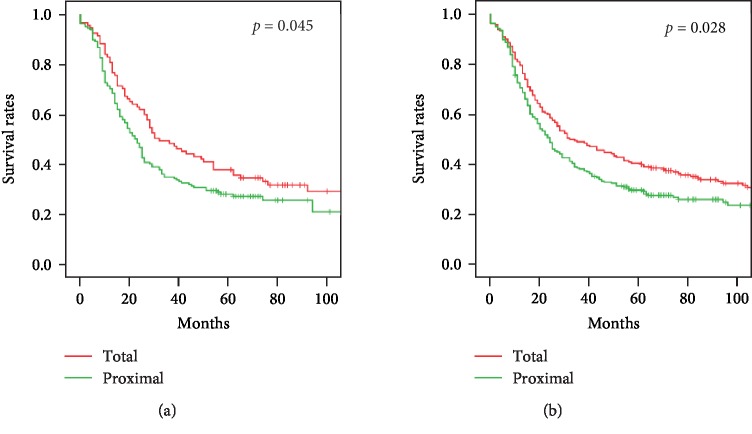
Survival of patients with upper gastric cancer and treated by total or proximal gastrectomy. (a) Invasion of the serosa, *p* = 0.045; (b) Borrmann type 3 or 4 infiltration, *p* = 0.028.

D2 total gastrectomy with 1–7, 8a, 9, 10, 11p, or 12a lymph node dissection and Roux-en-Y reconstruction were performed in 333 patients. D2 proximal gastrectomy with esophagogastrostomy was performed in 281 patients, ten with jejunal interposition, and 25 with double-tract reconstruction. The spleen was preserved unless it contained metastatic lymph nodes. Better survival rates were achieved with total gastrectomy than with proximal gastrectomy (*p* = 0.029, Figure 5). One-year survival of the 333 patients with D2 total gastrectomy was 81.3%, 3-year survival was 54.4%, and 5-year survival was 45.2%. One-year survival of the 316 proximal gastrectomy patients was 75.4%, 3-year survival was 44.9%, and 5-year survival rate was 36.7%. In patients with tumors that had invaded the serosa, survival was better with total than with proximal gastrectomy (*p* = 0.045, Figure 6(a)). Survival was also better with total than with proximal gastrectomy in patients with infiltrative type upper gastric cancer (*p* = 0.028, Figure 6(b)). Differences in survival were not associated with other patient characteristics (Table 7).

Twenty-one patients with total gastrectomy (6.3%) and 36 with proximal gastrectomy (11.4%) had postoperative complications. Five total gastrectomy patients experienced wound infections, three developed pneumonia, and two experienced fluid esophagitis; and intestinal obstruction, abdominal infection, and pancreatic fistula occurred in one patient each. There were seven occurrences of diarrhea, anemia, pneumothorax, and dumping syndrome. Wound infections and anastomotic leakage each occurred in eight proximal gastrectomy patients, five patients developed pneumonia, and four experienced reflux esophagitis. There were three cases of intestinal obstruction, three of abdominal infection, and two of pancreatic fistula. There were ten reports of diarrhea, anemia, pneumothorax, emphysema, and heart failure.

## 4. Discussion

The biological behavior of tumors determines the occurrence, development, and pathological features of tumors. It is a sign of the nature and malignancy of tumors. The gastric cancer in different parts has great differences in biological behavior. The macroscopic type, growth pattern, and degree of tissue typing of gastric cancer can correctly reflect the biological behavior of gastric cancer, and it is a sign of malignant accumulation and invasive expansion of gastric cancer.

Diagnosis of early stage disease has been reported in only 4.3% of patients with upper gastric cancer [13], which is significantly less frequent than in patients with lower gastric cancer. In this series, 7.2% of the upper gastric cancers were found at an early stage, which was significantly lower than the 21.4% of early lower gastric cancer diagnoses. The proportion of tumors that were ≥ 5 cm was significantly greater in patients with upper than with lower gastric cancer; more upper than lower gastric cancer tumors were poorly differentiated. The pT stage was significantly more advanced in upper than in lower gastric cancers; infiltration was deeper; and pN stage, proportion of tumors with lymphovascular emboli, and the proportion of tumors with serosal invasion were all higher. Recurrence and metastasis after surgery were also more frequent in patients with upper than with lower gastric cancer.

The study results are consistent with previous findings that survival is worse with upper than with lower gastric cancer. In this series, 5-year survival was 41.1% in upper gastric cancer compared with 57.2% in lower gastric cancer patients (Figure 1), which is comparable to the 40% 5-year survival reported by others [14, 15]. Univariate analysis showed that the tumor diameter, macroscopic type, pTNM stage, lymphovascular tumor emboli, serosal invasion, tumor recurrence and metastasis, and postoperative adjuvant chemotherapy all affected the prognosis of upper gastric cancer. Five-year survival was 50.9% when the tumor diameter was <5 cm and 34.1% when it was ≥5 cm; 77.9% for early and 38.0% for advanced disease; 51.4% for local and 34.2% for infiltrative tumors; 44.3% in the absence of and 31.4% in the presence of lymphovascular tumor emboli; 30.3% with and 47.5% without serosal invasion; 7.2% with and 66.2% without recurrence and metastasis; and 52.3% with and 36.0% without adjuvant therapy. Five-year survival also significantly decreased from 69.8% to 24.0% with the increase of pTNM stage from I to III. Multivariate analysis found that the macroscopic type, recurrence and metastasis, postoperative adjuvant chemotherapy, and pTNM stage were all independently associated with the prognosis of upper gastric cancer.

Most upper gastric cancers are diagnosed at a more advanced stage than lower gastric cancers; few cases of early disease are detected. The macroscopic type is generally infiltrative, the tumor diameter is large, and the tissue is poorly differentiated and has invaded the serosa. Lymph node metastasis, late pTNM stage, and recurrence and metastasis after surgery are the main contributors to a worse prognosis than that of lower gastric cancer. Adjuvant chemotherapy is known to increase the effectiveness of surgery in advanced gastric cancer [16–22]. Five-year survival was 52.3% in patients with adjuvant chemotherapy compared with 36.0% in those who did not receive it, which supports a recommendation for adjuvant chemotherapy in upper gastric cancer patients following resective surgery.

Lymph node metastasis has a poor prognosis in gastric cancer, which makes the removal of a sufficient number of regional lymph nodes an important component of radical surgery. Five-year survival was 28.7% in patients with and 63% in those without lymph node metastasis. The finding of lymph node metastasis in 64.3% of upper gastric cancer patients compared with 58.8% of those with lower gastric cancer is consistent with previous reports [23]. In this study, male sex, tumors ≥ 5 cm, poor tumor differentiation, macroscopic infiltration, and serosal invasion were more frequent in patients with lymph node metastasis. Statistical analysis found that sex, tumor diameter, macroscopic type, pT stage, tissue growth pattern, and serosal invasion were independent risk factors for lymph node metastasis of upper gastric cancer.

Gastric cancer recurs in situ or with lymph node, peritoneal, or liver metastasis or with hematogenous spread outside the liver with metastasis to other locations. Postoperative recurrence and metastasis may be related to tumor characteristics such as size, tissue type, differentiation, growth mode, pTNM staging, the extent of radical surgery, lymph node dissection, and intraoperative spread of cancer cells [24, 25]. Gastric cancer prognosis includes a high risk of recurrence and metastasis after radical gastrectomy [26]. Recurrence after radical gastrectomy is estimated to occur in approximately 50% [27], to more than 70% of patients with lymph node metastasis at diagnosis [28]. In this study, recurrence and metastasis occurred in 42.7% of upper gastric cancers and in 33.9% of lower gastric cancers. Recurrence and metastasis in patients with upper gastric cancer and preoperative lymph node metastasis were 54.3%, which is lower than previously reported. One-year survival of patients with postoperative recurrence and metastasis was 70.0%; the 3-year survival was 20.2%; and the 5-year survival was 7.2%, all of which were lower than the 83.6%, 71.5%, and 66.2% survival of patients without recurrence and metastasis, respectively. Recurrence and metastasis were more frequent in patients with tumors ≥ 5 cm, macroscopic infiltration, lymphovascular tumor emboli, serosal invasion, and increased pTNM stage. The macroscopic type, lymphovascular tumor emboli, and pTNM stage were independently associated with recurrence and metastasis.

Radical surgery is recommended by the Japanese Gastric Cancer Association Classification of Gastric Carcinoma fifteenth edition. D2 radical lymph node dissection is recommended, but differences of surgical procedures result in differences of the number of lymph nodes collected. It is still not clear whether total gastrectomy or proximal gastrectomy is preferred [29]. Total gastrectomy achieves sufficient resection and a wide range of lymph node dissection. Proximal gastrectomy preserves part of the stomach, allowing reconstruction of the digestive tract and better physiological recovery [30]. In upper gastric cancer surgery, dissection of lymph nodes 10 and 11b in the second station involves consideration of the spleen and possibly pancreatic body resection. However, two European randomized trials found that D2 surgery with spleen resection did not provide a survival benefit but rather increased the incidence of postoperative complications and mortality [11, 31]. A study by the Japan Clinical Oncology Group (JCOG0110) that compared splenectomy and spleen-preserving surgery did not find a significant difference in the 5-year survival in a group of over 500 gastric cancer patients (75.1% vs. 76.4%). The incidence of postoperative complications was higher in the splenectomy group (30.3%) than in the spleen-preserving group (6.7%, *p* < 0.05). Splenectomy should be avoided in patients with total gastrectomy for upper gastric cancer that does not involve the greater curvature and is without splenic lymph node metastasis. In such patients, splenectomy increases the occurrence of complications without increasing survival [32]. Neither the Japanese gastric treatment guidelines nor the National Comprehensive Cancer Network recommends prophylactic splenectomy [33]. The JCOG9501 study, which included patients who were at high risk, found an 8.4% incidence of No. 10 lymph node metastasis [34]. Whether splenectomy can improve survival in patients at risk of lymph node 10 metastasis because of the extent of local invasion, invasion of the greater curvature, or clinical suspicion of lymph node 10 metastasis deserves study.

### 4.1. Study Strengths and Limitations

The strengths include a relatively large sample size, which increased the statistical power. The retrospective design is a limitation, as the data may lack variables that could affect the results.

## 5. Conclusion

Upper and lower gastric cancer patients differed in sex, age, tumor diameter, tissue type, growth pattern, macroscopic type, infiltration depth, degree of lymph node metastasis, lymphovascular tumor emboli, and postoperative tumor recurrence and metastasis. Upper gastric cancers were more aggressive, had a worse prognosis, and were more prone to recurrence and metastasis after radical gastrectomy than lower gastric cancers. Surgical treatment of upper gastric cancer by radical total gastrectomy achieved better survival radical proximal resection.

## Figures and Tables

**Table 1:** Clinicopathological characteristics of patients with upper and lower gastric cancer.

	Upper gastric cancer (%)	Lower gastric cancer (%)	*p* value
	*n* = 649	*n* = 1551	
Sex			<0.001
Male	531 (81.8)	1098 (70.8)	
Female	118 (18.2)	453 (29.2)	
Age (years)			0.003
<60	303 (46.7)	833 (53.7)	
≥60	346 (53.3)	718 (46.4)	
Tumor diameter (cm)			<0.001
<5	267 (41.4)	946 (61.0)	
≥5	382 (58.9)	605 (39.0)	
Tissue type			0.046
Well differentiated	270 (41.6)	575 (37.1)	
Poorly differentiated	379 (58.4)	976 (62.9)	
Tissue growth mode			<0.001
Agglomerate type	131 (20.2)	434 (28.0)	
Nest type	185 (28.0)	467 (30.1)	
Diffuse type	333 (25.7)	650 (41.9)	
Macroscopic type			
Early	47 (7.2)	332 (21.4)	<0.001^∗^
Local (Borrmann 1, 2)	126 (19.4)	212 (13.7)	0.068^#^
Infiltrative (Borrmann 3, 4)	476 (73.4)	1007 (64.9)	
pT			<0.001
T1	47 (7.2)	331 (21.3)	
T2	67 (10.3)	318 (20.5)	
T3	271 (41.8)	527 (34.0)	
T4a	224 (34.5)	340 (21.9)	
T4b	40 (6.2)	35 (2.3)	
pN			0.041
N0	232 (35.7)	639 (41.2)	
N1	116 (17.9)	299 (19.3)	
N2	138 (21.3)	284 (18.3)	
N3a	110 (16.9)	235 (15.2)	
N3b	53 (8.2)	94 (6.1)	
Lymphovascular tumor embolus			0.034
Positive	172 (26.5)	346 (22.3)	
Negative	477 (73.5)	1205 (77.7)	
Serosal invasion			<0.001
Positive	264 (40.7)	375 (24.2)	
Negative	385 (59.3)	1176 (75.8)	
Recurrence and metastasis			<0.001
Positive	277 (42.7)	526 (33.9)	
Negative	372 (57.3)	1025 (66.1)	

^∗^Early versus advanced cancer; ^#^comparison of advanced cancers.

**Table 2:** Multivariate analysis of tumor characteristics and upper gastric cancer prognosis.

	*B*	SE	Wald	*p*	RR	95% CI
Macroscopic type	0.264	0.103	6.603	0.010	1.302	1.065–1.591
Recurrence and metastasis	1.547	0.112	190.958	<0.001	4.695	3.770–5.847
Adjuvant chemotherapy	−0.548	0.112	24.078	<0.001	0.578	0.464–0.719
pTNM stage	0.343	0.085	16.206	<0.001	1.410	1.193–1.666

*B*: beta coefficient; SE: standard error of the mean; Wald: Wald statistic; RR: risk ratio; CI: confidence interval.

**Table 3:** Patient characteristics and lymph node metastasis of upper gastric cancer.

	No metastasis	Metastasis	*p* value
	*n* = 231	*n* = 418	
Sex			0.011
Male	201	330	
Female	30	88	
Age (years)			0.936
<60	107	195	
≥60	124	223	
Tumor diameter (cm)			<0.001
<5	126	141	
≥5	105	277	
Tissue typing			0.005
Well differentiated	113	157	
Poorly differentiated	118	261	
Tissue growth mode			0.166
Agglomerate type	52	79	
Nest type	72	113	
Diffuse type	107	226	
Macroscopic type			
Early	39	8	<0.001^∗^
Local (Borrmann 1, 2)	65	61	<0.001^#^
Infiltrative (Borrmann 3, 4)	127	349	
pT			<0.001
T1	39	8	
T2	47	20	
T3	81	190	
T4a	55	165	
T4b	9	35	
Lymphovascular cancer embolus			0.180
Positive	54	118	
Negative	177	300	
Serosal invasion			<0.001
Positive	64	200	
Negative	167	218	
Recurrence and metastasis			0.188
Positive	113	227	
Negative	118	191	

^∗^Early versus advanced cancer; ^#^comparison of advanced cancers.

**Table 4:** Multivariate analysis of tumor characteristics and lymph node metastasis in upper gastric cancer.

	*B*	SE	Wald	*p* value	RR	95% CI
Sex	−0.433	0.206	4.419	0.036	0.648	0.433–0.971
Tumor diameter	0.570	0.166	11.837	0.001	1.768	1.278–2.447
Macroscopic type	0.639	0.157	16.610	<0.001	1.894	1.393–2.575
pT stage	0.739	0.170	18.833	<0.001	2.095	1.500–2.925
Tissue typing	0.442	0.147	9.075	0.003	1.556	1.167–2.074
Serosal invasion	−0.575	0.271	4.499	0.034	0.562	0.330–0.957

*B*: beta coefficient; SE: standard error of the mean; Wald: Wald statistic; RR: risk ratio; CI: confidence interval.

**Table 5:** Patient characteristics and recurrence and metastasis of upper gastric cancer.

	Recurrence and metastasis	No recurrence and metastasis	*p* value
	*n* = 277	*n* = 372	
Sex			0.246
Male	221	310	
Female	56	62	
Age (years)			0.987
<60	129	173	
≥60	148	199	
Tumor diameter (cm)			0.004
<5	96	171	
≥5	181	201	
Tissue typing			0.099
Well differentiated	105	165	
Poorly differentiated	172	207	
Tissue growth mode			0.473
Agglomerate type	58	73	
Nest type	72	113	
Diffuse type	147	186	
Macroscopic type			
Early	6	41	<0.001^∗^
Local (Borrmann 1, 2)	36	90	<0.001^#^
Infiltrative (Borrmann 3, 4)	235	241	
Lymphovascular cancer embolus			0.003
Positive	90	82	
Negative	187	290	
Serosal invasion			0.002
Positive	132	132	
Negative	145	240	
Adjuvant chemotherapy			0.412
Positive	186	261	
Negative	91	111	
pTNM stage			<0.001
I	16	74	
II	58	156	
III	203	142	

^∗^Early versus advanced cancer; ^#^comparison of advanced cancers.

**Table 6:** Multivariate analysis of tumor characteristics and recurrence and metastasis of upper gastric cancer.

	*B*	SE	Wald	*p* value	RR	95% CI
Macroscopic type	0.379	0.189	4.003	0.045	1.460	1.008–2.116
Lymphovascular tumor emboli	0.440	0.191	5.287	0.021	1.552	1.067–2.258
pTNM typing	0.966	0.149	42.105	<0.001	2.628	1.963–3.519

*B*: beta coefficient; SE: standard error of the mean; Wald: Wald statistic; RR: risk ratio; CI: confidence interval.

**Table 7:** One-, 3-, and 5-year survival of upper gastric cancer patients with total or proximal gastrectomy.

	Total gastrectomy				Proximal gastrectomy				*p*
	*n*	1 year (%)	3 years (%)	5 years (%)	*n*	1 year (%)	3 years (%)	5 years (%)	
	333	81.3	54.4	45.2	316	75.4	44.9	36.7	0.029
TNM stage									
I	37	84.9	71.7	65.9	53	94.6	83.8	75.4	0.385
II	79	86.7	60.0	53.3	135	81.0	69.4	59.3	0.305
III	217	74.2	41.4	28.1	128	68.7	29.5	24.1	0.112
Macroscopic type									
Early	22	96.0	88.0	79.1	25	90.9	86.4	76.4	0.350
Local	45	81.5	56.8	45.7	81	88.9	64.4	59.2	0.309
Infiltrative	266	79.5	49.0	40.0	210	70.7	38.2	29.2	0.028
Tumor diameter									
<5 cm	115	91.9	85.1	62.8	148	82.3	57.7	47.0	0.258
≥5 cm	214	78.0	47.0	36.9	168	70.1	37.9	31.0	0.194
Serosal invasion									
Positive	164	81.4	57.0	48.4	221	78.6	55.3	44.1	0.689
Negative	169	81.1	48.4	37.9	95	70.4	34.9	27.3	0.045

## Data Availability

This study data is from the “Department of Surgical Oncology, The First Affiliated Hospital of China Medical University, Shenyang (110001), China,” and from patients with gastric cancer who underwent R0 resection for gastric cancer at The First Affiliated Hospital of China Medical University from January 1980 to December 2012. Raw data cannot be provided for personal and commercial purposes.
